# Horizontal Transfers of Tc1 Elements between Teleost Fishes and Their Vertebrate Parasites, Lampreys

**DOI:** 10.1093/gbe/evs069

**Published:** 2012-08-09

**Authors:** Shigehiro Kuraku, Huan Qiu, Axel Meyer

**Affiliations:** ^1^Department of Biology, University of Konstanz, Germany; ^2^Konstanz Research School Chemical Biology, University of Konstanz, Germany; ^3^Present address: Genome Resource and Analysis Unit, Center for Developmental Biology, RIKEN, Kobe, Japan; ^4^Present address: Bigelow Laboratory for Ocean Science, West Boothbay Harbor, Maine

**Keywords:** lamprey, Salmonidae, Tc1 transposase, horizontal gene transfer

## Abstract

Horizontal gene transfer (HGT) has been recognized to be an important mechanism that shaped the evolution and genomes of prokaryotes and unicellular eukaryotes. However, HGT is regarded to be exceedingly rare among eukaryotes. We discovered massive transfers of a DNA transposon, a Tc1 element encoding a transposase, between multiple teleost fishes and lampreys that last shared a common ancestor over 500 Ma. Members of this group of Tc1 elements were found to exhibit a mosaic phylogenetic distribution, yet their sequences were highly similar even between distantly related lineages (95%–99% identity). Our molecular phylogenetic analyses suggested that horizontal transfers of this element happened repeatedly, involving multiple teleost fishes that are phylogenetically only distantly related. Interestingly, almost all the affected teleost lineages are also known to be subject to lamprey parasitism, suggesting that the horizontal transfers between vertebrates might have occurred through parasite–host interaction. The genomes of several northern hemisphere lamprey species, including that of the sea lamprey (*Petromyzon marinus*), were found to contain thousands of copies of the foreign elements. Impact of this event is discussed in relation to other peculiar genomic features of lampreys.

## Introduction

Vertical transmission of genetic information is the basis for evolution by common descent. Therefore, the composition of genomes would be expected to reflect the evolutionary history of their lineages. Incongruencies between gene and species trees are a tell-tale sign that unusual evolutionary mechanisms such as the horizontal movement of genes across evolutionary lineages, horizontal gene transfer (HGT), might have occurred. It is now recognized that foreign DNA elements can, albeit rarely, invade genomes and serve as, sometimes important, factors promoting lineage-specific genomic changes (Koonin 2009). Increasing availability of sequence information also from more and more nonmodel species now facilitates the discovery of genetic exchange among distinct lineages of vertebrates. Many of the discovered instances of horizontal transfers among vertebrates involved transposable elements (Kordis and Gubensek 1998; Leaver 2001; de Boer et al. 2007; Pocwierz-Kotus et al. 2007; Pace et al. 2008; Novick et al. 2010), and some of these cases are thought to have been mediated by retroviruses as vectors (Yohn et al. 2005; Tarlinton et al. 2006; Piskurek and Okada 2007). However, in none of these cases of genetic transfer among vertebrates were the actual mechanisms discovered.

In this study, we report an instance of frequent horizontal transfers of a DNA transposon, a Tc1 element encoding a transposase (Miskey et al. 2005), between lampreys and multiple teleost species, especially in fishes that are documented to be victims of lamprey parasitism. Some, but not all species of lampreys are known to be parasites of diverse fish (e.g., in the Great Lakes of North America). During the parasitic period of their life cycle, they attach to fish with their suction disk mouth ringed with sharp teeth and use their tongue to rasp through the scales and skin of a host to feed on its blood and body fluids (Hardisty and Potter 1971). Our results suggest that transfers of this DNA element to and from the lamprey genome occurred repeatedly and involved several teleost fish lineages that are not phylogenetically closely related but are known to be afflicted by lamprey parasitism. Hence, these horizontal transfer events are likely to have occurred through this parasite–host interaction.

## Materials and Methods

### Sequence Retrieval

Whole-genome assemblies of anole lizard, western clawed frog, zebrafish, fugu, green spotted pufferfish, medaka, and stickleback were downloaded from Ensembl (Hubbard et al. 2009). Sequences registered as annotated nucleotide, expressed sequence tag (EST), and genome survey sequence (GSS) of other actinopterygian fishes were downloaded from GenBank (as of April 2010). Vector sequences inside EST and GSS were masked using CROSS_MATCH (http://www.phrap.org/, cited 2012 Aug 29) using National Center for Biotechnology Information (NCBI) UniVector as reference. The masked sequences were partitioned between species and were assembled separately using CAP3 (Huang and Madan 1999). Sequences with significant overlaps (≥99% identity in ≥40 bp stretch) were assembled.

### *In Silico* Detection of Tc1 Elements

**Fig. 1.—:**
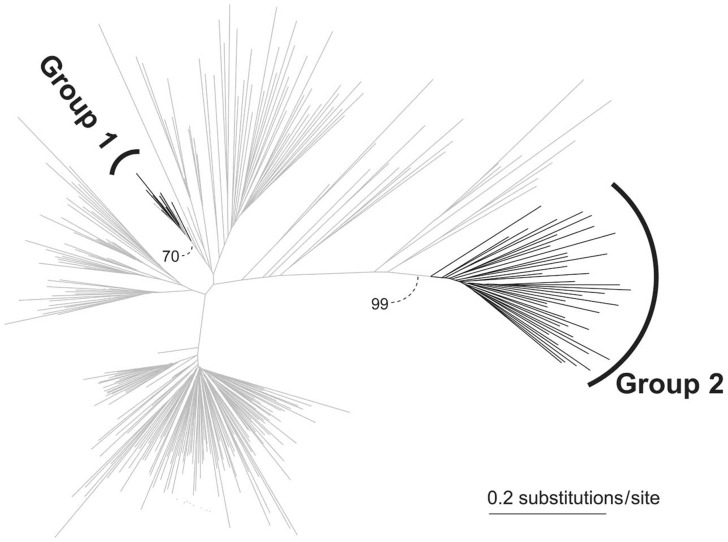
Molecular phylogenetic tree of vertebrate Tc1-like sequences. Lamprey sequences are shown in black, whereas Tc1 sequences from other genomes are shown in gray. The tree was inferred using 309 amino acid sequences (269 residues; see Materials and Methods). See Results and Discussion for designation of Group 1 and Group 2.

The nucleotide sequence of Atlantic salmon Tc1 element, previously designated DTSsa1 (EF685954), was used as a query for a BLASTN search of the sea lamprey PMAR3 genome assembly and assembled EST sequences. A consensus sequence for a subgroup of Tc1 elements of our interest (1,610 nt, supplementary fig. S1, Supplementary Material online) was generated from the top 300 blast hits using a simple majority rule as practiced previously (Pace et al. 2008) and was subsequently used as a query to search for closely related sequences in the aforementioned sequence data using BLASTN. Sequences of all hits that had at least 75% overlap in length with the open reading frame (ORF) in the consensus sequence were retrieved and aligned using MAFFT (31). To search more distantly related Tc1 sequences, the protein sequence encoded by the Atlantic salmon Tc1 element, DTSsa1, was used as a query in a tBlastn search against the whole-genome assemblies. Sequences with ≥30% identity in >20 amino acid stretch were retrieved, and the deduced peptide sequences were generated using GENEWISE (Birney et al. 2004). Most copies of Tc1 contain frame-shift or/and nonsense mutations disrupting ORFs. To utilize these sequences, which account for the majority of the Tc1 diversity, we deduced their peptide sequences by removing putative codons containing frame-shift or nonsense mutations and included them into the analysis together with those with intact ORFs (fig. 1).

### Phylogenetic Tree Reconstruction

**Fig. 2.—:**
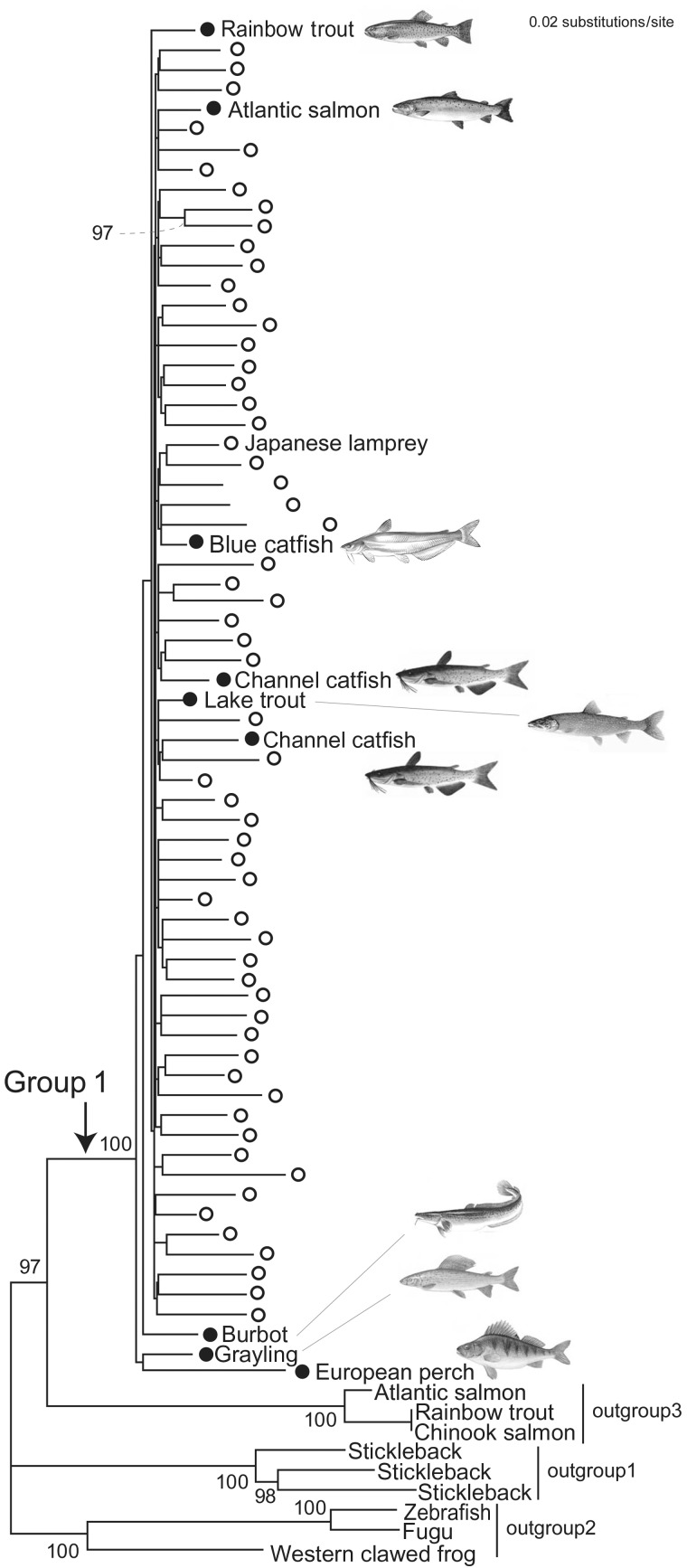
Molecular phylogenetic tree of Group 1 Tc1 sequences. This tree was inferred with the maximum-likelihood method based on 78 nucleotide sequences (1,020 sites). Highly similar sequences within species were excluded except for one (see Materials and Methods). White open circles indicate lamprey sequences, of which a sequence of the Japanese lamprey is particularly indicated. Filled circles indicate teleost sequences. Only bootstrap values greater than 80 are shown. Bootstrap values for remaining nodes are all below 67 (particularly, those within Group 1 are mostly less than 30). See supplementary table S1, Supplementary Material online, for details of outgroups 1–3.

To reduce computational load, we removed highly similar sequences (≥95% identity for nucleotide sequences; ≥80% for peptide sequences) among abundant data set for each species. The representative sequences were aligned using MAFFT (Katoh et al. 2005). Sites with gaps in ≥15% of aligned sequences and partial sequences shorter than 75% of the sequence alignment were removed. The neighbor-joining tree in figure 1 was inferred with MEGA4 (Tamura et al. 2007) using Poisson-corrected distance for amino acid sequences. The maximum-likelihood tree in figure 2 was inferred with RAxML (Stamatakis 2006) using nucleotide sequences based on the GTR + Γ model. Bootstrap probabilities were based on 1,000 replicates.

### Polymerase Chain Reaction

Genomic DNA of sea lamprey, Japanese lamprey, short-headed lamprey, pouched lamprey, Atlantic hagfish, inshore hagfish, elephant shark, beluga, Florida spotted gar, European chub, barbel, northern pike, European perch, grayling, stickleback, bullhead, an African cichlid, green spotted pufferfish, zebrafish, Atlantic salmon, African clawed frog, Japanese quail, thornback ray, lesser spotted dogfish, lake trout, medaka, soft-shelled turtle, Siamese crocodile, and mouse were extracted using phenol/chloroform (Sambrook and Russell 2001).

The genomic DNAs of the species mentioned earlier and those gifted were subjected to polymerase chain reaction (PCR) amplification. Three primer pairs were designed based on Group 1 Tc1 sequences: pair 1, 5′-CCAAGGATGTCAGGGACAAG-3′ and 5′-CCCAGTTGTCTTCTGAATCATTC-3′; pair 2, 5′-CACACTACGCCGTGAAGGAC-3′ and 5′-GCTGAGGGAAGGAGGTTCTC-3′; and pair 3, 5′-GCAGCTGGGACCATAGTCAC-3′ and 5′-GCTCCATTTTGGTCTCATCTGAC-3′. As a positive control, 18S ribosomal DNA was amplified using a forward primer 5′-AGCCTGAGAAACGGCTACCAC-3′ and a reverse primer 5′-GCTGCTGGCACCAGACTTG-3′. PCR was performed with PyroStart Fast PCR Master Mix (Fermentas) using a thermal cycler iCycler (BioRad) with the following condition: initial denaturation at 95°C for 1 min; 35 cycles of three steps, namely denaturation at 95°C for 1 s, annealing at 62°C for 5 s, and extension at 72°C for 15 s.

## Results and Discussion

### Identification of Tc1-Like Sequences in the Lampreys, Teleost Fishes, and Other Species

In two GenBank database entries for lamprey sequences, AF464190 and AB272083, we identified DNA regions that are highly similar to each other and that have a large number of almost identical matches in the ESTs of lampreys. These two stretches of lamprey DNA were found to be very similar (∼94% identity in aligned regions) to the DTSsa1 element of Atlantic salmon that encodes a Tc1-type of transposase (de Boer et al. 2007).

Our search in NCBI sequence database detected significant similarity of the aforementioned lamprey DNA elements to protein-coding sequences of the rainbow trout (AAN34802 and BAF37936) and to nucleotide sequences of the channel catfish (DQ400445) and the whitefish (GQ925661). Additional searches in assembled ESTs (see Materials and Methods) detected significantly similar sequences to this element in other teleosts including the northern pike, whitefish, and chinook salmon (table 1). We also identified highly similar sequences in the Asian blood fluke (*Schistosoma japonicum*) and even a protozoan fish parasite (*Ichthyophthirius multifiliis*) as well as in the sea louse (*Lepeophtheirus salmonis*), a parasite of fish, such as the Atlantic salmon (Krkosek et al. 2007) (table 1). Overall, among teleosts, we identified the largest number of sequences with significant similarity to the lamprey DNA elements in the Atlantic salmon. However, this is probably the case because of the abundant sequence resources for this species.

**Table 1:** Species with Group 1 Tc1 Sequences

Species	Accession ID^a^	Highest Identity^b^ (%)	Record of Lamprey Parasitism
Sea lamprey	FD709257^c^, FD710750^c^	97	Not applicable
Japanese lamprey	DC614714^c^, DC614669^c^	96	Not applicable
Atlantic salmon	DW582443^c^, DW568371^c^	98	Cudmore-Vokey and Crossman (2000) and Maitland (2003)
Chinook salmon	CB485379^c^	98	Cudmore-Vokey and Crossman (2000)
Lake whitefish	CB483831^c^, CB483902^c^	97	Cudmore-Vokey and Crossman (2000)
Lake trout	GU725401^d^, GU725399^d^	98	Cudmore-Vokey and Crossman (2000)
Rainbow trout	CX723061^c^, BX858799^c^	97	Cudmore-Vokey and Crossman (2000)
Grayling	GU725415^d^, FF847237^c^	96	—
Burbot	GU725394^d^, GU725397^d^	96	Scott and Crossman (1973)
European perch	GU725423^d^, GU725418^d^	92	Scott and Crossman (1973)
Northern pike	EV372296^c^, GH260996^c^	95	Scott and Crossman (1973)
Blue catfish	CK408358^c^	96	Cudmore-Vokey and Crossman (2000)
Channel catfish	CK412844^c^, CK416149^c^	96	Cudmore-Vokey and Crossman (2000)
Fish parasite, *Ichthyophthirius multifiliis*	EG962890^c^, EG965482^c^	98	—
Blood fluke, *Schistosoma japonicum*	BU712543^c^	96	—
Sea louse, *Lepeophtheirus salmonis*	ADND01366168^d^, ADND01125528^d^	98	—

^a^Accession IDs in NCBI database of up to two sequences with the highest similarities for each species.

^b^The highest sequence identity with the consensus Group 1 Tc1 sequence.

^c^Transcript sequences.

^d^Genomic sequences.

### Unique Phylogenetic Grouping Including Hosts and Parasites

Based on an alignment including sequences with ≥60% identity with any sea lamprey Tc1 at the amino acid sequence level, a molecular phylogenetic tree was constructed after discarding highly similar sequences within species (see Materials and Methods). This analysis divided sea lamprey Tc1 sequences into two distinct genetic clusters (fig. 1). The first cluster (designated “Group 1”) included the Atlantic salmon DTSsa1 and Tc1 from several other teleosts and the lampreys, whereas Group 2 consisted of only Tc1 sequences from the sea lamprey. Sea lamprey sequences in the Group 2 were much more divergent than those in the Group 1 (fig. 1). The overall topology of this phylogenetic tree was very different from the known phylogeny of the fish species from which they are derived (Chen et al. 2004; Steinke et al. 2006; Azuma et al. 2008; Li et al. 2008).

In an effort to investigate the relationships within the Group 1 Tc1 elements more closely, we reconstructed a phylogenetic tree that focuses on these elements alone (fig. 2). Group 1 not only comprised Tc1 elements mainly from the sea lamprey but also includes Tc1 elements from a diverse set of teleosts as well: from, for example, Atlantic salmon, channel catfish, blue catfish, grayling, lake trout, burbot, and European perch (fig 2). In fact, according to the species phylogeny, these teleosts with Group 1 Tc1 elements do not form a monophyletic group (Azuma et al. 2008; Li et al. 2008) and are placed taxonomically into four rather distantly related orders (Salmoniformes, Gadiformes, Siluriformes, and Perciformes). Moreover, Tc1 sequences from any particular species did not necessarily form a monophyletic group as is apparent for the sea lamprey, and relationships among teleosts did not match the species phylogeny (fig. 2).

To evaluate possible evolutionary scenarios more rigorously, we tested tree topologies supporting monophyly of the lamprey sequences or species phylogeny of teleosts and assessed them with the maximum-likelihood method (supplementary table S1, Supplementary Material online). Remarkably, likelihoods of those tree topologies in support of a vertical transmission were significantly lower than that of the tree topology in figure 2. Despite that internal relationships inside the Group 1 Tc1 sequences in figure 2 are hardly well resolved, this ML analysis and the extremely high sequence similarity (up to 98% at the nucleotide level; supplementary fig. S2, Supplementary Material online) support significant phylogenetic proximity among the lamprey and teleost Tc1 sequences and thus strongly argues for recent horizontal rather than vertical transmission.

The phylogenetic patterns suggested a particular scenario for the horizontal transmission of these DNA elements, in spite of the unclear position of the root of the tree (fig. 2). That is, the nodes before and after the origin of the Group 1 Tc1 elements are occupied by teleost sequences, suggesting that the initial horizontal transfer might have occurred from teleost fish to lampreys. It is also noteworthy that teleost sequences that are nested with clusters of lamprey Tc1 sequences are always derived from a single species (fig. 2). Such a phylogenetic pattern would best be explained by independent horizontal transfers from lamprey to ancestral lineages of those species with the Group 1 Tc1 elements. It is highly likely that the horizontal transfer was not a single unidirectional event—in the molecular phylogenetic tree (fig. 2), lamprey sequences are not nested in those of other species and vice versa. If the pattern in the phylogenetic tree is taken for granted, the complexity of the tree topology is therefore best interpreted as a consequence of sequential HGT events (Keeling and Palmer 2008).

Interestingly, there are records of parasitism by lampreys targeting all species included in Group 1 (table 1). In contrast, we did not detect Group 1 Tc1 sequence in any other vertebrate species with already sequenced genomes, such as anole lizard, zebrafish, medaka, stickleback, puffer fishes, chicken, frog, and mammals—and none of these species have ever been documented to being victims of lamprey parasitism. We therefore suggest that the Group 1 Tc1 elements have been horizontally transferred between lampreys and teleost fishes, mediated by an unknown “vector.” We note that the horizontal transfers might have involved fish parasites because only those species have Group 1 Tc1 elements outside vertebrates (table 1).

In some cases of HGTs, foreign elements have maintained basic sequence properties in their new donor genomes. Especially, our recent study suggested that the protein-coding genes of lampreys generally exhibit an extreme pattern of codon usage bias (Qiu et al. 2011), serving as a potentially reliable marker to identify foreign sequences and the direction of their transmission. However, codon usage bias in the sequences of the Group 1 Tc1 elements showed a unique pattern, which was different from those of both lampreys and teleost fishes (data not shown). Thus, the codon usage bias could, in this instance, not serve as a valid marker of the proposed event.

### Structure and Diversity of Group 1 Tc1 Elements

In the sea lamprey genome assembly (version PMAR3), our BLASTN search detected 6,604 copies of this element that were ≥90% identical (at the nucleotide level) to the Group 1 Tc1 consensus sequence (see Materials and Methods for our procedure to construct the consensus sequence). These sequences occupied ∼6.4 Mb of the 831.7 Mb (∼0.7%) of the lamprey genome assembly. In addition, 86 more divergent sequences (30%–50% at the amino acid sequence level; Group 2) were detected by more tolerant homology searches (see Materials and Methods).

**Fig. 3.—:**
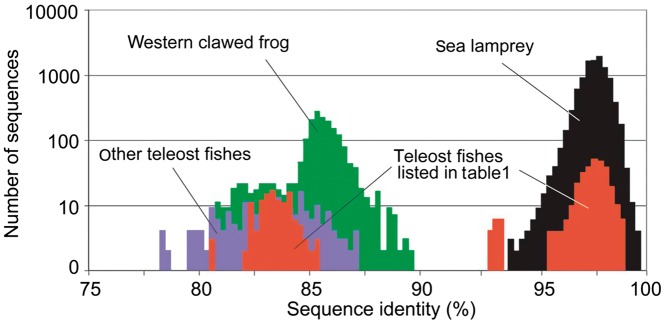
Similarity distribution of Tc1 sequences. Sequence identities were calculated in comparison with the Group 1 Tc1 consensus sequence (see supplementary fig. S1, Supplementary Material online, and Materials and Methods). Identities are based on nucleotide sequences. Colors indicate different species (or groups of species): black, sea lamprey; purple, zebrafish, medaka, fugu, *Tetraodon,* and stickleback; green, western clawed frog; and red, teleosts possessing Group 1 Tc1 elements in table 1. Only sequences with more than 150 bp of aligned sites were included. Note that Group 2 Tc1 sequences of the sea lamprey are not included because of lower identity.

We analyzed the distribution of sequence divergence within and between species, by computing percentage identity of available vertebrate Tc1 sequences to the Group 1 Tc1 consensus sequence (fig. 3). There is a clear division between Group 1 sequences (92.5%–100%) and other Tc1 sequences (<90%) in accordance with the unique grouping of lamprey and teleost sequences in the phylogenetic analysis (fig. 2). Most sea lamprey sequences were 97.5%–98.0% identical to the consensus sequence, and the peak for teleost Group 1 sequences was at a similar degree of divergence (fig. 3). This suggests that the intragenomic proliferation of these Group 1 Tc1 elements in lampreys and their teleosts’ host genomes coincided across species and that the intragenomic proliferation at least within the sea lamprey genome and the horizontal transfer involving the sea lamprey are not ongoing any more or not as active as before.

Our computation of numbers of synonymous and nonsynonymous substitutions (*K*_s_ and *K*_a_, respectively) for arbitrary pairs of Group 1 Tc1 sequences mostly produced *ω* values (*K*_a_/*K*_s_) close to one (data not shown), suggesting neutral evolution of these Tc1 sequences (Hurst 2002). We also found that ORFs of most Tc1-like sequences are interrupted by frame-shift and nonsense nucleotide substitutions. However, we also detected many transcripts of the Group 1 Tc1 elements in publicly available ESTs of the sea lamprey, the Japanese lamprey, and teleosts (table 1). Furthermore, we found one case of an apparent insertion of a Group 1 Tc1 element in a 3′ untranslated region of mRNA coding a hemoglobin homolog of sea lamprey (CO545064). Overall, in these lamprey species, Group 1 Tc1 elements seem to constitute a considerable fraction of their transcriptome. However, the frequent disruption of ORF and lack of signatures of functional constraint based on the *K*_a_/*K*_s_ ratio indicate that most of them are not capable of active transposition any more.

### Detecting Group 1 Tc1 Elements with PCR

**Fig. 4.—:**
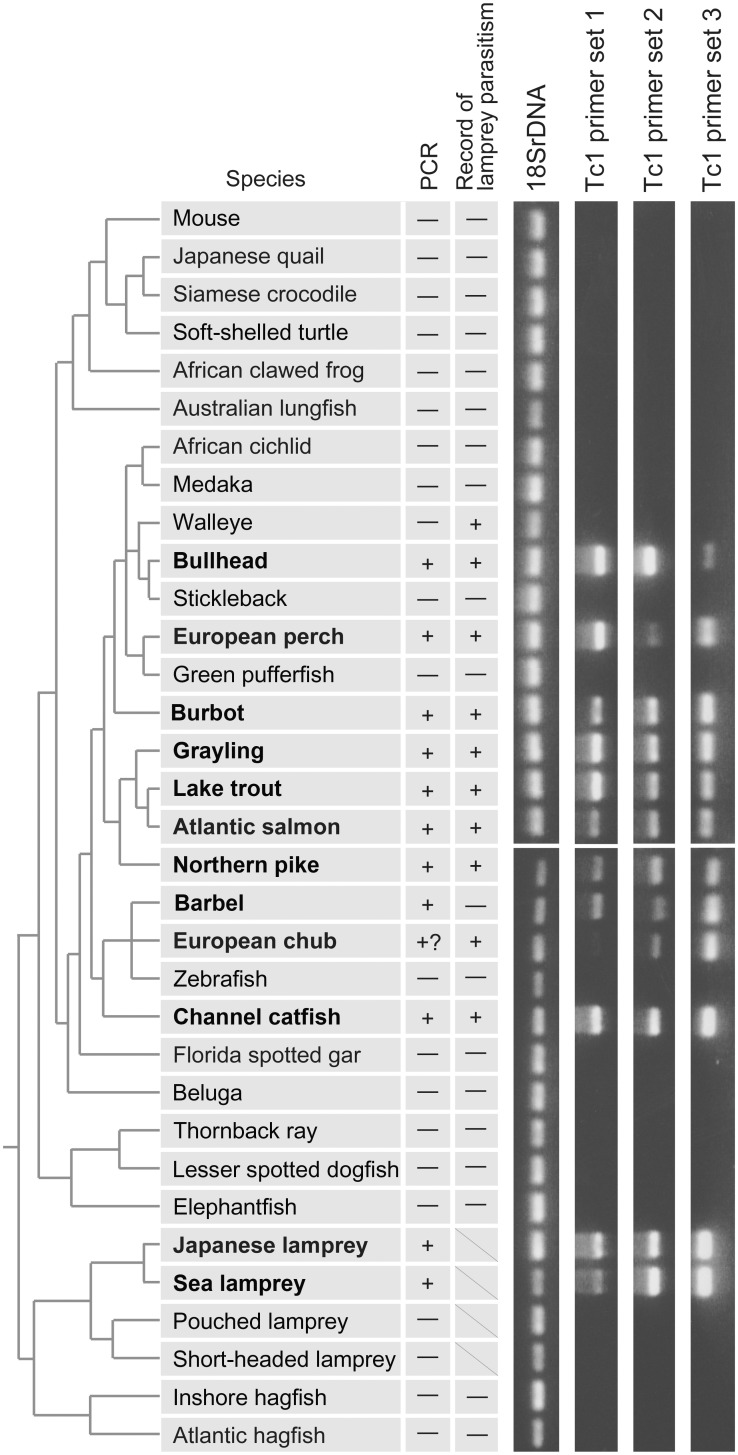
PCR amplification of Group 1 Tc1 elements in diverse vertebrates. Species phylogeny is based on molecular phylogenetic analyses (Inoue et al. 2003; Miya et al. 2003; Chen et al. 2004; Kikugawa et al. 2004; Kuraku and Kuratani 2006; Steinke et al. 2006; Mayden et al. 2007; Azuma et al. 2008; Li et al. 2008; Yagishita et al. 2009). Common names for species with positive PCR amplification of the Group 1 Tc1 elements are shown in bold. See supplementary fig. S3, Supplementary Material online, for species names and original gel images.

In an effort to characterize the phylogenetic distribution of the Group 1 Tc1 elements, we searched for them by PCR in the genomes of a diverse set of vertebrates (fig. 4). Three primer pairs, designed to amplify Group 1 Tc1 elements, resulted in positive bands in two northern hemisphere lampreys, *P. marinus* and *L. japonicum* (fig. 4). Except for the lampreys, amplification was detected only in some teleost species (fig. 4). Interestingly, in all species surveyed (except for the walleye) that are known to be parasitized by lampreys, we indeed identified Group 1 Tc1 elements (fig. 4).

No amplification of Group 1 Tc1 elements were detected for hagfishes, southern hemisphere lampreys, cartilaginous fish, and nonteleost actinopterygian fish (sturgeon and gar), as well as sarcopterygian species (fig. 4). In teleosts fishes, some species including all the five species with sequenced genomes did not show any amplification of Group 1 Tc1 elements in the genomic PCR (fig. 4), as has been suggested before by our in silico sequence searches (table 1). If the co-occurrence of the Group 1 Tc1 element in lampreys and the teleosts was simply derived from vertical transmission since the base of the vertebrate phylogeny, this scenario would have required at least six independent losses of all copies of the Group 1 Tc1 elements in lineages outside the Teleostei (hagfishes, southern hemisphere lampreys, cartilaginous fish, sturgeons, gars, and sarcopterygians) and several more losses within Teleostei. Importantly, the teleosts that do possess Group 1 Tc1 elements are not monophyletic (fig. 2) (Chen et al. 2004; Steinke et al. 2006; Azuma et al. 2008; Li et al. 2008). For these reasons, we conclude that the patchy phylogenetic distribution of the Group 1 Tc1 elements is best explained by their repeated horizontal transfers between northern hemisphere lampreys and multiple independent teleost lineages. The split between the northern hemisphere lamprey lineage and the southern hemisphere lineage is roughly estimated to be 200–100 Ma (Kuraku and Kuratani 2006). Our data therefore suggest relatively recent horizontal transfer events in the northern hemisphere lamprey lineage alone.

### Insights into Lamprey Ecology and Genome Biology

It is often documented that lampreys parasitize large fish (Hardisty and Potter 1971). However, small fish can also be attacked by lampreys (Cochran and Jenkins 1994). It is also known that in rare circumstances, lampreys can parasitize nonactinopterygian fish, such as sturgeon (Patrick et al. 2009), paddlefish (Hardisty and Potter 1971), gar (Hardisty and Potter 1971), and sharks (Wilkie et al. 2004), although physiological effects of the parasitism on much larger fish with dermal scutes may differ from those on teleosts. In addition to parasitism, predation can also be a route of horizontal transfer. For example, barbel (with positive PCR bands in fig. 4) feed on small lamprey. The descriptions and records of lamprey attacks may neither be necessarily accurate nor complete, and the actual routes of the transfers and species involved need to be carefully assessed by a larger taxon sampling.

The lamprey genome has been shown to exhibit several unusual characteristics: its karyotype consists of a large number of small chromosomes (*n* = 168 in *P. marinus*; Potter and Rothwell 1970), programmed genomic rearrangement (Smith et al. 2009), an unusual codon usage bias, and amino acid composition (Qiu et al. 2011) as well as high GC content in protein-coding regions (Kuraku 2008). We show that as much as approximately 0.7% of the lamprey genome is composed of the elements that are derived from parasite–host horizontal transfers. These remarkable genomic characteristics that had not been documented so far in any other vertebrates emphasize the uniqueness of the evolution of the lamprey genome. More surprising findings may surface in de novo sequencing and comparative genomic studies covering more diverse evolutionary lineages.

## Conclusions

The uncovered surprising similarity of Tc1 elements that are shared between the genomes of lampreys and multiple teleost fish lineages suggests several bouts of recent HGT between them. The in silico and taxonomic–experimental investigations of this element in diverse vertebrates provided strong evidence that species that are known to be hosts of lamprey parasitism, except one case, possess this element. These data sets therefore both support the scenario that the co-occurrence of these sequences was mediated by lampreys. The genomes of northern hemisphere lamprey species, such as the sea lamprey, carry thousands of copies of the DNA elements, and their expansion was likely caused by or facilitated through parasite–host interaction.

## Supplementary Material

Supplementary table S1 and figures S1–S3 are available at *Genome Biology and Evolution* online (http://www.gbe.oxfordjournals.org/).
